# Effect of early granulocyte-colony-stimulating factor administration in the prevention of febrile neutropenia and impact on toxicity and efficacy of anti-CD19 CAR-T in patients with relapsed/refractory B-cell lymphoma

**DOI:** 10.1038/s41409-021-01526-0

**Published:** 2022-01-30

**Authors:** Raphaël Liévin, Roberta Di Blasi, Florence Morin, Eugenio Galli, Vincent Allain, Romain De Jorna, Laetitia Vercellino, Nathalie Parquet, Miryam Mebarki, Jerome Larghero, Eric de Kerviler, Isabelle Madelaine, Sophie Caillat-Zucman, Sylvie Chevret, Catherine Thieblemont

**Affiliations:** 1 APHP, Hôpital Saint-Louis, Service Hémato-Oncologie, Paris, France; 2 DMU-DHI, Université de Paris – Paris Diderot, Paris, France; 3 APHP, Hôpital Saint-Louis, Laboratoire d’immunologie, Pari – Université de Paris, Paris, France; 4 Sezione di Ematologia, Dipartimento di Scienze Radiologiche ed Ematologiche, Università Cattolica del Sacro Cuore, Roma, Italy; 5 APHP, Hôpital Saint-Louis, Service de Pharmacie, Paris, France; 6 APHP, Hôpital Saint-Louis, Service de médecine nucléaire, Paris, France; 7 APHP, Hôpital Saint-Louis, Aphérèse, Paris, France; 8 APHP, Hôpital Saint-Louis, Thérapie cellulaire, Paris, France; 9 APHP, Hôpital Saint-Louis, Service de radiologie, Paris, France; 10 APHP, Hôpital Saint-Louis, Biostatistiques, France – Université de Paris – Paris Diderot, Paris, France

**Keywords:** Immunotherapy, B-cell lymphoma, B-cell lymphoma

## Abstract

Chimeric Antigen Receptor T cells (CAR-T) are an outbreaking treatment option for relapsed/refractory (R/R) diffuse large B-cell lymphoma (DLBCL). Cytokine release syndrome (CRS) and immune effector cell-associated neurotoxicity syndrome (ICANS) are the most common specific toxicities, while severe neutropenia and infections are often observed as well. From March 2020, early G-CSF prophylaxis at day (D) two post-infusion was systematically proposed. We then compared patients treated before that date who did not receive G-CSF or who received late (after D5) G-CSF as control group. Patients administered with early G-CSF had similar duration of grade 4 neutropenia but significantly decreased incidence of febrile neutropenia (58% versus 81%, *p* = 0.018). Similar rate of toxicities was observed, including overall and grade 3-4 CRS (*p* = 0.93 and *p* = 0.28, respectively), and overall and grade 3-4 ICANS (*p* = 0.62 and *p* = 0.88, respectively). We observed no difference in the quality of CAR T-cells expansion (*p* = 0.79, %Cmax), nor in response rate (best ORR, 57.6% vs 61.8%, *p* = 0.93), nor survival even in a group of patients adjusted by a propensity score. In conclusion, early G-CSF administration was safe and effective in reducing febrile neutropenia without impact on toxicities nor on anti-lymphoma activity of CAR-T.

## Introduction

In recent years, consistent progress was made in treating relapsed/refractory diffuse large B-cell lymphomas (R/R DLBCL) [1–4]. CAR-T cells are autologous T-cells with an engineered T-cell receptor, which artificially targets surface tumoral antigen. This approach is on active use since June 2018 in Europe for R/R B-cell malignancies, after pivotal Phase I-II studies of anti CD19 CAR-T cells were published [1–3]. Currently commercially available CAR-T are Tisagenlecleucel (Tisa-cel), indicated in R/R DLBCL and acute lymphoblastic leukemia, Axicabtagene-ciloleucel (Axi-cel), authorized for R/R DLBCL, primary mediastinal B cell lymphoma (PMBL) and transformed follicular lymphoma (tFL) and more recently Lisocabtagene-maraleucel (Liso-cel) allowed in the USA for R/R DLBCL. In accordance to first results of pivotal trials, a recent meta-analysis showed that for R/R DLBCL the overall response rate (ORR) and complete response rate (CRR) of CAR-T-cell therapy was 63% and 39%, respectively [5].

Safety profile of CAR-T involves well-established toxicities, as CRS and ICANS. CRS is a systemic inflammatory response provoked by soluble mediators with wide clinical spectrum, ranging from mild flu-like syndrome to severe shock. Besides CRS, ICANS is the other described complication, possibly involving an endothelial toxicity and causing variable neurologic signs, from slightly impaired cognitive function to coma or fatal seizures [6]. Both CRS and ICANS have been objected of a consensus for harmonizing gradings [7]. Hematologic toxicities can represent early and long-lasting complication of CAR-T and there is growing interest on their genesis and management. In 2019 Fried et al. described 72% severe neutropenia and 28% severe thrombocytopenia in 29 responding patients affected by R/R B-cell malignancies treated by CAR-T [8]. In this study, cytopenias mostly occurred with a biphasic pattern. The first nadir of cytopenias seemed to be related to effects of lymphodepletion and activity of CRS, possibly mediated by macrophages; this could not explain the second occurrence of cytopenias observed at day 40 or more. As described for patients treated with Rituximab, a role for perturbation in stromal-derived factor 1 (SDF-1)/CXCligand 12 (CXCL12), a chemokine implicated in regulation of hemopoietic stem cells migration and activity, has been proposed [8]. As this chemokine has active role also in B-cell developing, possible interference has been hypothesized for normal granulocyte recovery [9]. A CAR-Hematotox was recently defined, and a score ≥2 was used for predicting early severe neutropenia before 14 days after CAR T-cells infusion [10].

In this setting, there is no clear consensus about the role and safety of G-CSF administration. G-CSF has shown high efficacy in reducing neutropenia, febrile neutropenia and infections when used as prophylaxis in patients affected by lymphomas undergoing chemotherapy [11, 12]. On the other side, in vivo studies found an important role of monocyte/macrophage axes on CRS onset and that GM-CSF is key CRS-promoting protein involved in cytokine storm. Artificial inactivation of GM-CSF, via antibodies or genetic technologies, showed not to impact on anti-tumor capacity and proliferation of CAR-T in vitro, and to reduce secretion of CRS biomarkers depending from macrophages, including monocyte chemoattractant protein 1 (MCP-1), interleukin (IL) 6, and IL-8 [13, 14].

Only few data exist concerning the practical role and acquired risk of using G-CSF in CAR-T treated patients. We recently reported the safety of late G-CSF administration after day 5 in a series of 70 patients with R/R aggressive LBCL compared to patients with no G-CSF support [15]. Although we did not observe any impact of the incidence of grade 3-4 neutropenia or febrile neutropenia, the administration of late G-CSF after CAR-T infusion allowed reducing the duration of hospitalization without any impact on the toxicities, CRS or neurotoxicities, as well as on the efficacy of CAR-T in terms of response, examined by the outcome and on the CAR-T expansion. The objective of the present study was then to evaluate the efficacy of the G-CSF with an early and prophylactic administration at day 2 after the CAR T-cells infusion on the duration of severe neutropenia, the incidence of febrile neutropenia, and the prevalence of early infections in patients with R/R LBCL treated with CAR-T: with this aim, we compared the group of patients receiving the G-CSF at day 2 (early G-CSF) to the group of patients without G-CSF (no G-CSF) before March 2019, and the group of patients with G-CSF between March 2019 and March 2020 treated with late G-CSF after Day 5 (late G-CSF), as control group. The secondary objectives were to assess if early G-CSF in CAR-T treated patients could be considered safe when focusing on CAR-T specific toxicities (CRS, ICANS), and on the possible impact on the efficacy of CAR-T in terms of expansion, response rates and survivals (PFS and OS).

## Patients and methods

### Population

The study population was identified as all consecutive 122 patients presenting R/R aggressive LBCL treated in our center with commercialized anti-CD19 CAR-T Tisagenlecleucel or Axicabtagene-ciloleucel between June 2018 and November 2020. G-CSF administration started in March 2019 with a late administration after day 5 and demonstrated no impact on CAR T-cells safety and efficacy [15]. Hence, in March 2020, we began systematic G-CSF prophylaxis at day 2 post-infusion. Two groups were identified based on G-CSF administration:1/ the group “early G-CSF” in which patients had systematic prophylaxis G-CSF administration at day 2 from March 2020 including 33 patients; and 2/ the control group including the patients treated before March 2020 and who did not receive G-CSF in the 10 days following CAR-T infusion or that received a late administration of G-CSF between day 5 and 10 after CAR T-cells infusion. All patients but 4 received antipneumocystic prophylaxis with either cotrimoxazole (112), atovaquone (5) or pentamidin (1); 119 patients (98%) received antiviral prophylaxis using valaciclovir. None of the patients received prophylaxis for bacterial infections, as recommended by the EBMT [16].

### CAR-T treatment and G-CSF administration

**Fig. 1:**
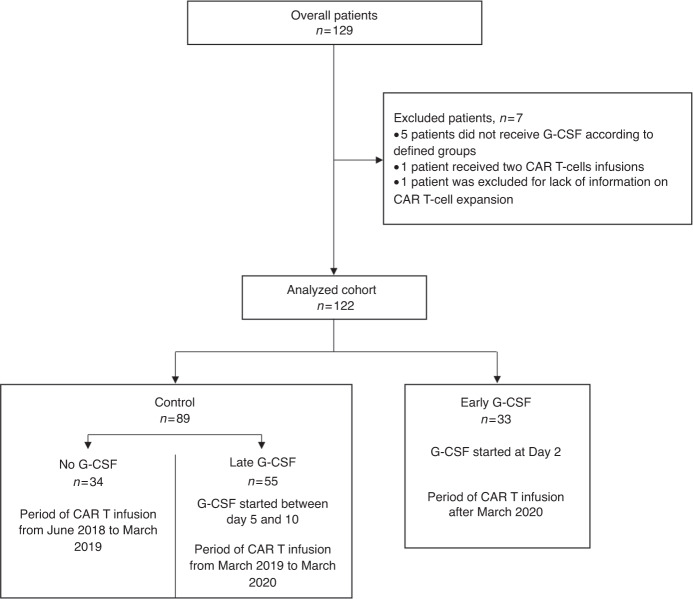
Flow chart of the analyzed patients. Distribution of study population.

Lymphodepletion consisted on a fludarabine-cyclophosphamide regimen administered from D-5 to D-3 with doses depending on specific commercial CAR-T product (daily doses were 25 mg/m^2^ + 250 mg/m^2^ for Tisa-cel and 30 mg/m^2^ + 500 mg/m^2^ for Axi-cel). Anti-CD19 CAR-T were infused at D0. G-CSF administration consisted in daily 30 or 48 MUI subcutaneous filgrastim injections (for patients with body weight < or ≥80 kg, respectively), pursued as long as grade 3 neutropenia persisted. From June 2018 to March 2019, no G-CSF was administered. Between March 2019 and March 2020, G-CSF was administered between day 5 to day 10 if neutropenia grade 4 persisted, otherwise no G-CSF was administered. From Mach 2020, prophylactic G-CSF was administered at day 2 (Fig. 1).

### Data collection

Clinical and biological data, together with dates of beginning of lymphodepletion and CAR-T infusion and outcome were collected. For each patient we registered the complete data at baseline, at time of bridging and treatment, and the status of disease before lymphodepletion and at fixed timepoints, imperatively assessed by PET-CT. Histology was confirmed by expert pathologists (VM) while PET-CT were standardized by an expert nuclear medicine physician (LV). CAR-T expansion by flow cytometry was implemented and has been described previously [15]. Data were retrospectively collected from November 2020 to January 2021. Missing data, mostly due to insufficient or lost to follow up, are displayed in each table.

### Primary endpoints evaluation

Primary endpoints were duration of severe neutropenia defined as grade 4 (neutrophils < 0.5 G/L), incidence of febrile neutropenia (defined as fever >38.3 °C once or twice >38 °C within 1 h associated with a grade 4 neutropenia) and incidence of documented infections occurring in the first 30 days after CAR-T reinfusion («early infections») in comparing the groups “early G-CSF” compared to a control group including the “no G-CSF” and “late G-CSF” patients. Neutropenia and infections were graded according to Common Terminology Criteria for Adverse Events (CTCAE 5.0).

### Secondary endpoints evaluation

The secondary endpoints included persistent cytopenia at one month and three months. Regarding safety profile, we targeted possible interactions between G-CSF and CAR-T in terms of occurrence and severity of CRS and ICANS, graded according to the American Society for Transplantation and Cellular Therapy (ASTCT) Consensus and compared according to CAR-T product. We also measured and compared between the groups the CAR T-cells expansion. CAR-Hematotox score including five baseline variables (platelet count, neutrophil count, hemoglobin, C-reactive protein, ferritin) was calculated and patients were considered high-risk if the score was ≥2 [10].

### Statistical analysis

Summary statistics, namely median with inter-quartile range (IQR) or percentages were used to describe patient characteristics across treatment groups.

Exact Fisher test and nonparametric Wilcoxon rank sum tests were used to compare groups defined at baseline, unless specified otherwise. Cumulative incidence of grade 4 neutropenia and early infections were estimated and compared through the Gray test. Persistent cytopenia at three months were evaluated using a landmark analysis based on patients alive at three months; comparison across G-CSF groups of CRS and ICANS were based on the stratified chi-square test where stratification factor was the CAR-T product.

Analyses were performed on R (https://www.R-project.org/). All tests were two-sided with *p* values of 0.05 or less denoting statistical significance.

No correction of *p* values for multiplicity of tests was performed so that results should be taken cautiously as possibly false positive.

Analyses of CAR-T cells expansion were performed on GraphPad Prism 5.0 statistical software package (San Diego, CA). Data were compared using Two-way analysis of variance followed by a Bonferroni test.

## Results

### Baseline characteristics of the patients

A total of 129 patients received commercialized CAR T-cells (axicabtagene-ciloleucel – Yescarta® - or tisagenlecleucel – Kymriah®) and 122 patients fulfilled the inclusion criteria (Fig. 1). Main patient and disease characteristics are summarized in Table 1. Median age was 60 [21–77], 29 (23.7%) were over 70 years old. At enrollment, most of the patients (*n* = 110, 90.1%) presented with a good PS 0 or 1, and 75.4% had a primary refractory disease. Median time between apheresis and infusion was 39 (range, 29–90) days. Between apheresis and lymphodepletion, 104 (85.2%) patients received a bridging therapy. This bridging therapy was classified as low regimen for 22 (18%) patients, including rituximab, rituximab-dexamethasone, brentuximab vedotin, or revlimid; one patient received radiotherapy and one patient corticosteroids alone; or as high regimen including ifosfamide-etoposide ±rituximab, ifosfamide-cyclophosphamide-etoposide (ICE)± brentuximab vedotin, gemcitabine-oxaliplatin (GEMOX)-rituximab, ICE-rituximab, or vinblastine. At the end of bridging, 77 (63%) patients were evaluated with progressive disease and 19 (15.6%) had stable disease. At time of treatment, IPI was scored as high in 22 (18%), and revised IPI as poor in 51 (41.8%) patients.

**Table 1:** Characteristics of overall population and according to ITT (interquartile range).

		Early G-CSF *n* = 33		Control *n* = 89		*p*
		*n*	Statistics	*n*	Statistics	
Age	Median (IQ)	65	(53–70)	60	49–68	0.12
	>70	11	33%	18	20.2%	0.13
Sex	Male	17	51.2%	58	65.2	0.21
	Female	16	48.5%	31	34.8	
Histology	DLBCL	24	72.7%	73	82	0.47
	PMBL	3	9.1%	5	5.6	
	tFL	6	18.2%	11	12.4	
DLBCL COO	GC	10	41.7%	37	52.9	0.48
	ABC	14	58.3%	33	47.1	
Refractory	Yes	23	69.7%	69	77.5	0.48
	No	10	30.3%	20	22.5	
Previous lines		2	2-2	3	2-4	<0.0001
Previous ASCT/		7	21%	26	29%	0.49
Previous AlloSCT		0	0%	0	0%	1
At enrollment						
PS	0	11	33.3%	40	44.9	0.5
	1	18	54.6%	41	46	
	2	4	12.1%	8	9	
Elevated LDH		24	72.7%	42	47.2	0.021
Ann Arbor stage	III or IV	27	81.8%	69	77.5%	0.60
Extranodal sites	≥2	11	33%	27	30.3%	0.75
R-IPI	Very good	0	0%	6	6.74%	0.059
	Good	13	53.9%	48	53.93%	
	Poor	35	39.3%	35	39.33%	
IPI	0	0	0%	6	6.74%	0.10
	I	6	18.2%	28	31.46%	
	II	7	21.2%	20	22.47%	
	III	13	39.4%	21	23.6%	
	IV	7	21.2%	10	11.24%	
	V	0	0%	4	4.49%	
At lymphodepletion						
PS	0	7	21.2%	43	48.3%	0.047
	1	17	51.52%	32	36%	
	2	6	18.2%	11	12.4%	
	3	3	9.1	3	3.37%	
Elevated LDH		17	51.52%	27	30.34%	0.051
Ann Arbor stage	III or IV	26	78.8%	70	78.7%	0.98
Extranodal sites ≥2		11	33.3%	28	31.4%	0.84
Status at lymphodepletion	Partial response	7	21.2%	19	21.3%	0.98
	Stable disease	5	15.2%	14	15.7%	0.93
	Progression	21	63.6%	56	62.9%	0.94
R-IPI	Very good	1	3%	10	11.24%	0.068
	Good	13	39.4%	47	52.8%	
	Poor	19	57.6%	32	36%	
IPI	0	1	3%	10	11.2%	0.12
	I	7	21.2%	22	24.7%	
	II	6	18.2%	25	28.1%	
	III	10	30.3%	19	21.4%	
	IV	9	27.3%	10	11.2%	
	V	0	0%	3	3%	
Prophylaxis	Antipneumocystis	32	96.7%	86	96.6%	1
	Antiviral	33	100%	86	96.6%	0.56
Bridging	No bridging	5	15.2%	13	14.6%	0.94
	Low	6	18.2%	21	23.6%	0.52
	High	22	66.6%	55	61.7%	0.62
CAR T product	Tisagencecleucel (kymriah)	15	45.5%	44	49.4%	0.69
	Axicabtagene-ciloleucel (yescarta)	18	54.4%	45	50.6%	
At infusion						
Neutrophils	Median (IQ), G/L	0.990	(0.41–2.06)	1.22	(0.62–1.992)	0.45
Hemoglobin	median (IQ) g/L	9.5	(8.6–10.9)	9.8	8.8–11.2	0.5
Platelets	Median (IQ), G/L	140	(87–195)	141	97–190	0.75
CRP	>30 mg/L	14	42.2%	26	29.2%	0.16
Albumin	<35 g/L	12	36.3%	17	19.1%	0.046
Lymphocytes	Median (IQ) G/L	0.02	(0–0.03)	0.04	(0.02–0.06)	0.002
Ferritine	median(IQ)	763	(414–1084)	721	(361–1128)	0.67
LDH	>Nl	17	51.5%	27	30.3%	0.036
Hemotox score	≥2	21	94%	75	84.3%	0.35

Considering these baseline characteristics, few differences appeared between the two groups, with a lower number of previous lines, a LDH level more elevated at time of decision and worse PS at time of lymphodepletion in the early G-CSF group compared to the control group (*p* = 0.0001, *p* = 0.012, *p* = 0.045, respectively).

### Cytopenias and infections before D30

**Fig. 2:**
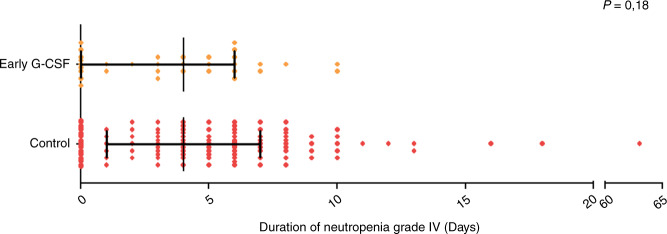
Duration of grade 4 neutropenia in group early G-CSF (D2) and in the control group including no G-CSF and late G-CSF after D5. Duration of grade 4 neutropenia between Early G-CSF (yellow) and Control (red) did not differ between groups (*p* = 0.18).

During the first 30 days after infusion, 95 (77.8%) patients presented an episode of grade 4 neutropenia, with no difference in incidence and duration between early G-CSF and control group (73% vs 80%, *p* = 0.46, and 5 vs 4 days, *p* = 0.18, respectively)(Table 2) (Fig. 2).

**Table 2:** Neutropenia and infections during the procedure (day 0–day 30) according to G-CSF administration.

Day 0–Day 30	Early G-CSF		Control		*p*
	*n*	%	*n*	%	
Grade IV neutropenia (<0.5 G/L)					
Prevalence d0–30, *n* (%)	24	73%	71	80%	0.46
Duration (d), median (range)	4 (0–6)	–	5 (2–8)	–	0.18
Duration of G-CSF administration during D0–D30 median (range)	5 (4–6)	–	2 (0–5)	–	0.28
Febrile neutropenia					
Prevalence D0–D30, *n* (%)	19	57.6%	72	80.9%	0.018
Early infection (d0–30) *n* (%)	11	33.3%	26	29.2%	0.66
Bacterial, *n* (%)	10	30.3%	18	20.22%	0.33
Viral, *n* (%)	4	12.1%	14	15.7%	0.78
Fungal, *n* (%)	1	3%	2	2.25%	1

Febrile neutropenia occurred in 91 (74.6%) patients, including 19 (57%) in the early G-CSF group, 72 (80.9%) in the control group (*p* = 0.018). Thirty-seven (30%) patients developed early infections defined by a documented infection, including 11 (33.3%) in the early G-CSF group and 26 (29.2%) in the control group (*p* = 0.66). This included 28 bacterial infections, 18 viral infections, and 3 fungal infections.

### Persistent cytopenias at day 30 and at day 90

Median follow-up was 10.4 months after CAR T-cells infusion.

**Fig. 3:**
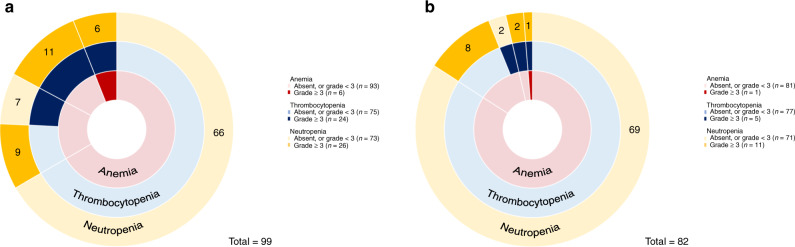
Prevalence of late cytopenias after CAR T-cell infusion. Sunburst chart showing the overlapping prevalence of neutropenia, thrombocytopenia, and anemia across at day 30 (**a**) and day 90 (**b**) post–CAR T infusion. Concentric rings are organized in a hierarchical structure outward from the origin, with overlapping segments representing shared cytopenias within the same patient(s); the numbers shown in each outer ring segment represent patient(s) with that unique combination of cytopenias. The prevalence of individual cytopenias are shown in the accompanying keys.

At day 30, 99 (81.1%) patients were alive without receiving additional treatment. Among these, prevalence of grade 3-4 cytopenia, including neutropenia, anemia, and thrombocytopenia, was 26%, 6%, 24% respectively, with no difference between the early G-CSF group and the control group (Table 3 and Fig. 3a).

**Table 3:** Cytopenia (neutropenia, anemia, thrombocytopenia) grade ≥3 at day 30 and day 90.

	All		early G-CSF		Control		*p*
	*n*	%	*n*	%	*n*	%	
Cytopenia at day 30							
Neutropenia G ≥ 3	26	26%	9	39%	17	19.1%	0.17
Anemia G ≥ 3	6	6%	3	13%	3	3.4%	0.14
Thrombocytopenia G ≥ 3	24	24%	7	30%	17	19.1%	0.42
Cytopenia at day 90							
Neutropenia G ≥ 3	11	13%	2	10%	9	10.1%	1
Anemia G ≥ 3	1	1%	1	5%	0	0%	0.24
Thrombocytopenia G ≥ 3	5	6%	3	16%	2	2.2%	0.09

At day 90, 82 patients were alive without receiving additional treatment and were evaluable for grade 3-4 persistent cytopenias (Fig. 3b). No difference was observed between the early G-CSF group and the control group.

### CRS and ICANS

Prevalence of any grade or grade ≥2 CRS occurred in 88 (72.2%), in 37 (30.3%) patients respectively, with no evidence of difference across groups (*p* = 0.88 and *p* = 1.00) (Table 4). Prevalence of any grade ICANS, and grade ≥2 ICANS occurred in 32 (26.2%) and 16 (13.1%) patients, again with no difference across groups (*p* = 1.00 and *p* = 0.37). Use of Tocilizumab and corticosteroids did not differ between groups (*p* = 0.73 and *p* = 0.71, respectively), nor did the ICU referral (*p* = 0.90) (Table 4).

**Table 4:** CRS and ICANS all and according to G-CSF administration.

	All		Early G-CSF		Control		*p*
	*n*	%	*n*	%	*n*	%	
Any grade *n* (%)	89	73%	24	72.7%	65	73%	1
CRS gr 1 *n* (%)	51	41.8%	10	30.3%	38	42.7%	0.21
CRS gr 2 *n* (%)	40	32.8%	13	39.4%	27	30.3%	0.34
CRS gr ≥3 *n* (%)	1	0.8%	1	3%	0	0%	–
Any grade *n* (%)	32	26%	9	27%	23	25%	0.37
ICANS gr 1 *n* (%)	16	13%	3	9.1%	13	14.6%	0.42
ICANS gr 2 *n* (%)	11	9%	5	15.2%	6	6.8%	0.15
ICANS gr ≥3 *n* (%)	5	4%	1	3.03%	4	4%	0.71
Tocilizumab (any administration)	27	22%	8	24%	19	21%	0.73
Tocilizumab > 1 dose	10	8.1%	4	12%	6	6.7%	0.33
Corticosteroid (any administration)	21	17%	5	15%	16	18%	0.71
Admission to the ICU	38	31%	10	30.3%	28	31.4%	0.90

### Impact of G-CSF administration on CAR-T expansion

**Fig. 4:**
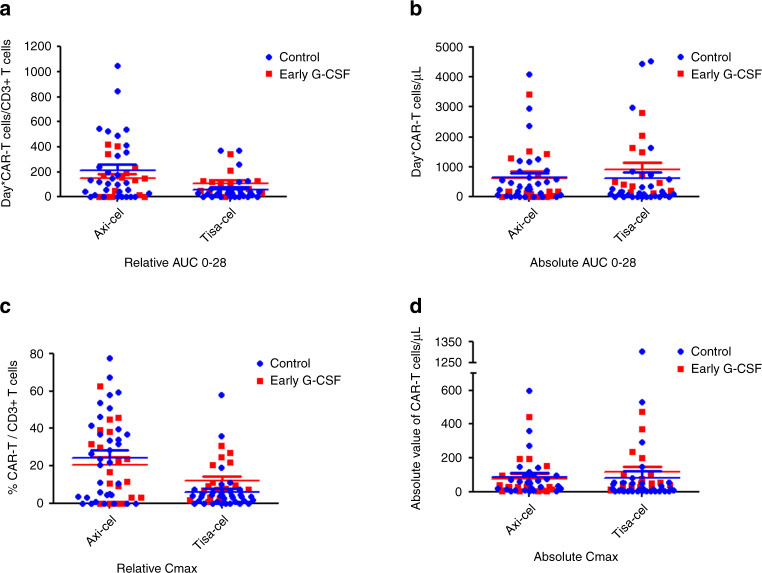
Expansion of CAR-T after injection, monitored by flow cytometry. Blue points: Control group; Red points: Early G-CSF group. **a** Relative AUC 0–28 calculated with R (https://www.R-project.org/) and for patients treated with Axi-cel or Tisa-cel and with at least 3 points of CAR-T expansion follow-up. **b** Absolute AUC 0–28 calculated with R and for patients treated with Axi-cel or Tisa-cel and with at least 3 points of CAR-T expansion follow-up. **c** Relative Cmax (maximum concentration detected in % CAR-T/CD3+ T cells) for patients treated with Axi-cel or Tisa-cel. **d** Absolute Cmax (maximum concentration detected in number of CAR-T cells/µL) for patients treated with Axi-cel or Tisa-cel.

As routine monitoring of CAR-T expansion was implemented some months after the beginning of CAR-T therapy in our institution, only a subgroup of patients could be studied for CAR-T pharmacokinetics in the G-CSF group compared control group. Patients treated with Tisa-cel and Axi-cel were analyzed separately due to different patterns of expansion between the two products. As shown in Fig. 4a–d, no difference was observed in the expansion pattern of CAR-T for Tisa-cel and Axi-cel treated patients both in term of relative and absolute quantification. Integrative pharmacokinetics parameters were used to better compare both groups for each CAR-T product. For Tisa-cel patients, relative AUC 0–28 (Days*CAR-T/CD3+ T cells) and absolute AUC 0–28 (Days*CAR-T cells /µL) showed no significant differences between early G-CSF and the control group (median relative AUC 0–28: 99 (IQR 39–126) *vs* median 29 (IQR 1–60) respectively, *p* = 0.83; median absolute AUC 0–28: 468 (IQR 232–1589) *vs* 99 (IQR 2–466) respectively, *p* = 0.59). Similarly, for Axi-cel patients, relative AUC 0–28 and absolute AUC 0–28 showed no significant differences between early G-CSF and the control group (median relative AUC 0–28: 144 (IQR 0–233) vs 115 (IQR 15–354) respectively, *p* = 0.83; median absolute AUC 0–28: 166 (IQR 0–1265) vs 331 (IQR 35–854) respectively, *p* = 0.59).

Likewise, for Tisa-cel patients, no significant differences between early G-CSF and the control group were found in median relative Cmax (% CAR-T/CD3+ T cells) (median 16.5 (IQR 3.1–38.3) vs median 22.7 (IQR 3.7–40.0) respectively, *p* = 79) and in median absolute Cmax (number of CAR-T cells /µL) (median 26 (IQR 5–139), vs median 55 (IQR 13–98) respectively, *p* = 0.75). Moreover, no significant differences between early G-CSF and the control group were found for Axi-cel patients in median relative Cmax (median 9 (IQR 4–22) vs median 4 (IQR 1–6) respectively, *p* = 0.79) and median absolute Cmax (median 50 (IQR 32–177) *vs* median 13 (IQR 2–49) respectively, *p* = 0.75).

### Incidence and grade of toxicities (cytopenias, ICANS, CRS) according to axicabtagene-ciloleucel (Yescarta) or tisagenlecleucel (Kymriah)

Before day 30, 78% patients presented an episode of grade 4 neutropenia (Supplementary Table 1), including 57 (90%) after axi-cel and 38 (64%) after tisa-cel. Febrile neutropenia was observed after in 55 (87.3%) patients after axi-cel and 36 (61.0%) after tisa-cel.

At day 30, incidence of grade 3-4 neutropenia, grade 3-4 anemia, and grade 3-4 thrombopenia was 11 (24%), 3 (6%) and 10 (22%) respectively in patients treated with Tisa-cel and 15 (28%), 3 (5%) and 14 (26%) respectively in patients treated with Axi-cel, (*p* = 0.13, *p* = 1.00, *p* = 0.30, respectively).

At day 90, incidence of grade 3-4 neutropenia, grade 3-4 anemia, and grade 3-4 thrombocytopenia according to CAR-T product was 6 (18%), 1 (3%) and 2 (6%) respectively in patients treated with Tisa-cel and 5 (10%), 0 (0%), 3 (6%) respectively in patients treated with Axi-cel, (*p* = 0.14, *p* = 0.40, *p* = 1.00).

Incidence of any grade CRS, grade ≥2 CRS and median duration of CRS was respectively 42 (71%), 15 (25%) and 5,5 (2–7) days for patients receiving Tisa-cel and 46 (73%), 22 (35%) and 6 (4–10,8) for patients receiving Axi-cel (*p* = 0.84, *p* = 0.32, *p* = 0.06) respectively.

Incidence of any grade ICANS, grade ≥2 ICANS was respectively 9 (15%), 1 (1%) for patients receiving Tisa-cel but 23 (36%), 15 (24%) and for patients receiving Axi-cel (*p* = 0.013, *p* = 0.0002) respectively. Median duration did not differ according to the product (*p* = 0.94).

### Score Hematotox and predictive factors of neutropenia

At baseline, Hematotox score was calculated for 121/122 patients. Hematotox score was ≥2 in 106 (86.6%) of patients, 31 (93.9%) in the early G-CSF group and 75 (85.2%) in the control group (*p* = 0.35). In the 106 patients with baseline Hematotox score ≥2, grade IV neutropenia and febrile neutropenia occurred in 87 (82.1%) and 80 (75.4%), respectively. In those 84 patients with baseline Hematotox score ≥ 2 exposed at day 30, incidence at day 30 of grade 3-4 neutropenia, grade 3-4 anemia, and grade 3-4 thrombocytopenia was 23 (21.6%), 6 (5.7%) and 23 (21.6%) respectively, with no difference between the G-CSF groups (*p* = 0.09, *p* = 0.16, *p* = 0.57). In the 71 patients with baseline Hematotox score ≥2 exposed at day 90, incidence at day 90 of grade 3-4 neutropenia, grade 3-4 anemia, and grade 3-4 thrombocytopenia was 9 (12.7%), 1 (1.4%) and 5 (7.0%) respectively, with no difference between the G-CSF groups (*p* = 1.00, *p* = 0.27, *p* = 0.12)

Univariate analysis selected the Hematotox score for neutropenia before day 30 and for neutropenia at day 30, but not for neutropenia at day 90 (*p* < 0.0001, *p* = 0.018, *p* = 0.20 respectively). Tisa-cel was also selected for neutropenia before day 30 only (*p* = 0.001). Multivariate analysis selected Hematotox score and tisa-cel for neutropenia before day 30.

### Efficacy of CAR-T

Responses to CAR-T therapy were stated with PET-CT at M1, M3, M6, M9, and every year. Complete response rate at 1 month and 3 months in the group early G-CSF compared to the control group were 37% and 33% (*p* = 0.79) and 55% and 58% (*p* = 0.91), respectively. No difference was observed when comparing the two groups considering the response rate at M1 (*p* = 0.11) and at M3 (*p* = 0.91), nor when considering OS (*p* = 0.70) and PFS (*p* = 0.20).

## Discussion

In this report, we observed that in a series of patients with R/R DLBCL treated with CAR-T, early administration of G-CSF was associated with reduced febrile neutropenia without any impact on the toxicities, CRS or neurotoxicities, as well as on the efficacy and expansion of CAR-T in term of response.

This small retrospective series of patients showed clinical and biological characteristics comparable to general epidemiology of R/R DLBCL [1–3]. Patients treated after March 2020 presented more advanced disease (elevated LDH) and less median previous lines compared to the historical group: As LDH unbalance may be stochastic, the lower number of previous lines mirrors the deepened CAR-T culture of the referral centers which allows to treat patients at an optimized early timepoint.

In a meta-analysis of 2005, G-CSF administration for chemo-induced febrile neutropenia has been associated with shorter hospitalization and more rapid neutrophils recovery [17]. We considered as parameters of efficacy of G-CSF administration the duration of severe neutropenia (<0.5 G/L), and the prevalence of febrile neutropenia, and early infections. In our observation, no difference was observed in duration of severe neutropenia, but the incidence of febrile neutropenia was significantly decreased with early G-CSF, and this seems the most important clinical outcome. Deeper neutropenia was observed in early G-CSF group without reaching statistical significance. We assume here that the toxicity of the fludarabine-cyclophosphamide conditioning regimen was higher for patients in early G-CSF group, which presented more advanced disease and slightly older age. At the same time, our incidence of grade 4 neutropenia is comparable to what is described in real life experiences, were grade 3 and 4 neutropenia in the first month from CAR-T is reported in 95% patients by Jain et al. [18], and in 98% and 73% of cases, for grade ≥3 and grade 4 respectively by Baird et al. [19]. In the previous study, G-CSF was administered in 62% patients within month 6; in the latter, G-CSF was used uniquely beyond day 28 post infusion and was administered in 51% patients to maintain ANC > 1000 G/L.

Patients receiving early G-CSF tended to be more neutropenic at D0 and had more advanced disease: this may lead to hypothesize a more elevated risk for infections, despite few literature available for lymphoproliferative diseases [20, 21]. Thus, the absence of difference in prevalence of early infections between the two groups may be considered as a harmonization of exceeding risk of the group early G-CSF. Baird et al. reported 46–55% early infections, mostly bacterial within the first month. These data are comparable to those reported in our statistics and fit with the high incidence rate of deep neutropenia. Usually, infections are not severe [19].

The safety of G-CSF administration during CAR-T treatment has been discussed: on the one hand, some consider the possibility for G-CSF to empower cytokine release and somehow worsen CAR-T specific toxicities, notably CRS and ICANS [14]. Jain et al. described deeper CRS and ICANS in patients with uncomplete blood counts recovery at 1 months, while no difference on cytokine peaks were registered, with the exception of macrophage derived chemokine (MDC) which was higher in the cytopenic cohort. Nevertheless, no information is available about G-CSF timing in patients with cytopenias, thus not allowing to postulate any causal relationship with MDC elevation, CRS or ICANS [18]. In our real-life cohort, 73% patients experienced any grade CRS, and there was no difference in prevalence nor in the grading when receiving early G-CSF or no or late GCSF. These numbers are in concordance with what is observed with the major CAR-T registrational studies JULIET, ZUMA-1, and TRANSCEND [1–3]. ICANS occurred in 26% of patients, most frequently of grade 0–2, with only 10% patients experiencing severe ICANS. All severe neurotoxicities were reversible, with two patients still experiencing mild word-finding deficits six months after CAR-T infusion. Notably, no difference in prevalence nor in grading of ICANS was observed when comparing patients receiving early G-CSF or no or late G-CSF.

As far as timing of G-CSF is concerned, no systematic data are available. In 2019 Gaut et al. presented a retrospective report describing 22 patients, uniquely treated with Axi-cel, among who 7 received G-CSF, with a non-harmonized timing for starting the administration and most patients receiving G-CSF from D0 to D5 [22]. All patients of that report received prophylactic tocilizumab 36 h after CAR-T infusion. They found no difference in prevalence of any grade CRS according to G-CSF administration. With regards to neurotoxicity, they report 50% overall patients experiencing any grade ICANS, mostly severe, with no difference according to G-CSF, as we describe in our cohort.

In G-CSF patients we observed the same quality of response at M1 and M3 evaluation over the two groups of patients. Moreover, we observed no difference in PFS and OS according to G-CSF administration. This was precisely confirmed when examining OS and PFS with the propensity score where characteristic of both groups were identical and comparable to those reported in pivotal trials.

In conclusion, this is at our knowledge the largest real-life study discussing the use of G-CSF in R/R DLBCL patients treated with CAR-T. We observed that administering G-CSF from D2 as prophylaxis in neutropenic patients was associated with reduced risk of febrile neutropenia without increasing the risk of severe CRS nor ICANS. G-CSF was safe also in preserving CAR-T expansion kinetics and anti-lymphoma activity, with no impact on the quality of response and outcomes. Therefore, routine G-CSF administration can be safely considered in the context of CAR-T treatment for R/R LBCL.

## Supplementary information

Table 5. (or Supplementary Table 1)

## Data Availability

For data, please contact catherine.thieblemont@aphp.fr.
