# Supplementary material for: Effect of early granulocyte-colony-stimulating factor administration in the prevention of febrile neutropenia and impact on toxicity and efficacy of anti-CD19 CAR-T in patients with relapsed/refractory B-cell lymphoma
Source: Bone Marrow Transplant. 2022 Jan 30;57(3):431–9. doi: 10.1038/s41409-021-01526-0 (PMC8907072; doi:10.1038/s41409-021-01526-0)
Supplement: Supplementary file 1 — Table 5. (or Supplementary Table 1) [file 41409_2021_1526_MOESM1_ESM.docx]

**Table 5. (or Supplementary Table 1): Neutropenia and infections during the procedure (d0 – d30), cytopenia grade > 3 at day 30 and day 90, CRS and ICANS according to CAR-T product**

|  | Axi-cel (Yescarta) | | Tisa-cel (Kymriah) | | p |
| --- | --- | --- | --- | --- | --- |
|  | n | % | n | % |  |
| **Day 0 – Day 30 Grade IV neutropenia (<0,5 G/L)** |  |  |  |  |  |
| **Prevalence d0-30, n (%)** | 57 | 90% | 38 | 64 | 0,00053 |
| **Duration (d), median (range)** | 4 (1-7) | - | 3 (0-6) | - | - |
| **Duration of G-CSF administration during D0-D30 median (range)** | 3 (0-5) | - | 3 (0-5) | - | - |
| **Febrile neutropenia** |  |  |  |  |  |
| **Prevalence D0-D30, n (%)** | 55 | 87,3% | 36 | 61% | **0,00086** |
| **Early infection (d0-30) n (%)** | 21 | 33,3% | 16 | 27,1% | 0,45 |
| **Bacterial, n (%)** | 14 | 22,2% | 14 | 23,7% | 0,84 |
| **Viral, n (%)** | 13 | 20,6% | 5 | 8,4% | 0,058 |
| **Fungal, n (%)** | 1 | 1,5% | 2 | 3,3% | 0,52 |
| **Late cytopenias** |  |  |  |  |  |
| **Neutropenia G>3** | 15 | 28% | 11 | 24% | 0,13 |
| **Anemia G>3** | 3 | 5% | 3 | 6% | 1 |
| **Thrombocytopenia G>3** | 14 | 26% | 10 | 22% | 0,30 |
| **Neutropenia G>3** | 5 | 10% | 6 | 18% | 0,14 |
| **Anemia G>3** | 0 | 0% | 1 | 3% | 0,40 |
| **Thrombocytopenia G>3** | 3 | 6% | 2 | 6% | 1 |
| **CRS and ICANS** |  |  |  |  |  |
| **Any grade CRS n (%)** | 46 | 73% | 42 | 71% | 0,84 |
| **CRS gr 1 n (%)** | 24 | 38% | 27 | 45,7% | 0,39 |
| **CRS gr 2 n (%)** | 21 | 33,3% | 15 | 25,4% | 0,33 |
| **CRS gr > 3 n (%)** | 1 | 1,5% | 0 | 0 | - |
| **Median duration of CRS (days)** | 6 | 4-10,8 | 5,5 | 2-7 | 0,06 |
| **any grade ICANS n (%)** | 23 | 36% | 9 | 15% | 0,013 |
| **ICANS gr 1 n (%)** | 8 | 12,6% | 8 | 13,5% | 0,88 |
| **ICANS gr 2 n (%)** | 10 | 15,8% | 1 | 1,6% | 0,0062 |
| **ICANS gr > 3 n (%)** | 5 | 7,9% | 0 | 0 | - |
| **Median duration of ICANS (days)** | 4 | 2-9,5 | 4 | 2-7 | 0,94 |
